# Quantifying carbon fluxes in pigs at different production stages: Intake, excretion, and retention

**DOI:** 10.1371/journal.pone.0355493

**Published:** 2026-08-11

**Authors:** Elvira Sattarova, Saman Lashkari, Henry J.H. Jørgensen, Frederik Rask Dalby, Christian Friis Børsting

**Affiliations:** 1 Department of Animal and Veterinary Sciences, Aarhus University, AU Viborg – Research Centre Foulum, Tjele, Denmark; 2 Department of Biological and Chemical Engineering, Aarhus University, Aarhus, Denmark; University of Life Sciences in Lublin, POLAND

## Abstract

A detailed understanding of how dietary carbon (C) is partitioned between excretion, gaseous emissions, and retention is essential for integrating C into current nutrient accounting frameworks, thereby bringing nutrient losses and gas emissions within a single standardized system. To support such integration, the present study aimed to construct C balance models for weaned pigs, grower–finisher pigs, and sows using extant prediction equations and established constants to estimate dietary C input and C outputs via excreta, gas emissions, and retention in body tissues. Predicted C balances showed variable agreement across production stages, with comparatively closer agreement observed in grower–finisher pigs. Estimates of C intake and C output via exhalation were generally consistent with reported values, whereas predictions of fecal, urinary, and enteric CH_4_ outputs showed greater deviation, indicating areas requiring further refinement. Overall, the models developed in this study offer a conceptual framework for incorporating C into a unified nutrient accounting system. The present study demonstrates the feasibility of combining existing equations to estimate C flows at the animal level, while also highlighting key uncertainties and data gaps that need to be addressed in future research.

## Introduction

Environmental concerns associated with livestock production systems, particularly in areas characterized by high animal population densities, have intensified markedly over recent years [1,2]. Pork is the second most consumed meat globally [3]. Yet, pig production is also the second-largest contributor to global anthropogenic greenhouse gas (GHG) emissions released from the livestock sector [4]. Beyond GHG emissions, pig production imposes a substantial environmental load due to excessive application of manure to soils. Due to nitrogen (N) and phosphorus (P) losses from fields, this practice contributes to the acidification and eutrophication of both terrestrial and aquatic ecosystems [5,6]. The environmental load associated with excessive manure application arises when there is an imbalance between the N and P application rates from manure and the nutrient requirements per hectare of cultivated land [6,7].

In Denmark, environmental protection legislation is enforced through the Danish manure normative system, which quantifies the nutrient content of livestock manure to facilitate annual fertilizer planning and thereby regulate its application on a per-hectare basis [8]. The Danish manure normative system relies on mass balance calculations of N, P, and potassium by accounting for inputs (feed and bedding), outputs (milk, eggs, and hair), and retention in animal growth (meat and fetuses), and the remainder is excreted by the animals in the form of urine and feces [8]. However, the current Danish manure normative system does not incorporate carbon (C) turnover, which is a prerequisite for the accurate quantification of methane (CH_4_) and carbon dioxide (CO_2_) from livestock production. Integrating C fluxes into the Danish manure normative value system would enable a more precise and transparent assessment of the on-farm C cycle, allowing mineral and gaseous emissions to be incorporated into a unified normative system. The aim of the study is to provide a theoretical basis for the on-farm C cycle by establishing C mass balances for weaned pigs, grower-finisher pigs, and sows, based on predicted dietary C inputs and C outputs via excretion, gas emissions from respiration and enteric processes, and retention in animal products.

## Materials and methods

The C mass balance is calculated as the difference between C inputs derived from diet composition, dietary nutrient content, feed intake, nutrient digestibility, and animal performance, and C outputs, including nutrient excretion in urine and feces, losses due to exhalation and enteric fermentation, and nutrient retention in the animal’s body. A C mass balance was calculated for weaned (6.4–31 kg), grower-finisher pigs (31–115 kg), and sows including piglets from birth until weaning. For sows, the C mass balance was estimated over an annual production cycle by proportionally weighting the gestation and lactation phases according to their respective durations (in days), multiplied by the number of litters per year to account for the full annual production cycle.

### Carbon input

A C mass balance was estimated based on the Danish national average diet composition for weaned and grower-finisher pigs and sows, as reported by the Danish research and development organization SEGES Innovation (Table 1) [9]. For grower-finisher pigs and sows, the C mass balance was estimated based on the national Danish averages for both homegrown and factory-produced diets. In the case of weaned pigs, the C mass balance was calculated using the mean values derived from homegrown and factory-produced diets, in accordance with the aggregated form in which the input data were provided. The diets described herein were used as input values for this study to test if the model can estimate the distribution of C within reasonable boundaries; however, the methodological approach is not limited to these diets and can be applied to other dietary formulations.

**Table 1:** Danish national average feed composition and calculated chemical composition of home-grown or factory-produced feed for growing pigs, finishing pigs, and sows.

	Pig category				
Item^a^	Weaned pigs	Grower-finisher pigs		Sows	
Feed production type	Average^2^	Home-grown	Factory-produced	Home-grown	Factory-produced
**Feed ingredients, %**					
Barley	26.1	40.0	27.0	40.0	31.0
Benzoic acid	0.44	0	0	0	0
Blood plasma	0.03	0	0	0	0
Calcium carbonate	1.18	1.44	1.44	1.45	1.43
Calcium formate	0.36	0	0	0	0
Cane molasses	0.50	0.10	1.00	0.20	1.00
Faba beans	0.64	1.0	2.0	0	0
Fish meal	0.10	0	0	0	0
Lard	0.41	0	0	0.20	0
Lysine, HCl	0.21	0	0	0	0
Lysine sulfate	0.60	0.60	0.57	0.27	0.28
Methionine	0.20	0.06	0.05	0.02	0.02
Monocalcium phosphate	0.91	0.42	0.22	0.60	0.45
Oat	0.82	0	0	1.5	1.5
Palm oil	0.41	0.15	0.78	0.20	0.78
Potato protein	1.23	0	0	0	0
Rapeseed cake	0.64	0	0	0	1.0
Rapeseed meal	0	0	1.0	0	0
Rye	0.90	10.0	10.0	9.0	12.0
Sodium chloride	0.52	0.40	0.40	0.40	0.40
Soybean meal, toasted	16.9	14.8	9.0	11.0	7.2
Soybean oil	0.90	0.15	0.10	0.56	0.50
Soy protein concentrate	1.44	0	0	0	0
Sugar beet pulp	0	0	0	2.50	3.50
Sunflower meal	0.64	1.5	7.0	1.0	5.0
Threonine	0.24	0.17	0.14	0.05	0.07
Tryptophan	0.07	0	0.01	0	0
Valine	0.10	0	0	0	0
Vitamin/mineral premix with phytase	0.22	0.22	0.22	0.22	0.22
Wheat	42.2	27.1	36.7	29.8	29.7
Wheat bran	0	0.30	2.45	0.50	4.0
Whey powder	0.70	1.60	0	0.50	0
FU per kg diet^b^	1.10	1.05	1.04	1.05	1.03
**Calculated chemical composition, g kg**^**-1**^ **DM**					
DM (g kg^-1^ feed)	858.8	864.3	858.7	860.6	859.4
Ash	65.7	53.6	52.7	53.3	54.5
CP	202.8	175.7	174.3	152.4	153.1
Crude fat	43.0	29.3	34.8	36.1	40.2
cTF	185.9	202.9	214.9	214.8	237.3
Starch	458.4	494.0	487.2	508.4	479.1
Sugar	39.4	44.8	36.4	34.7	36.3

^a^FU = Feed units; DM = dry matter; CP = crude protein; cTF = calculated total fiber; calculated as the difference between dietary organic matter content and dietary crude protein, crude fat, starch, and sugar.

^b^Average of home-grown and factory-produced feed composition over an annual production cycle.

Dietary nutrient concentration was calculated based on the nutrient concentration supplied by individual feed ingredients according to the following equation:


$$\begin{array}{c} X_{ik}=\;\sum_{j}^{n_{k}}a_{jk}x_{ij} \end{array}$$ (1)

where $X_{ik}$ where *i* = 1, 2, …, 9 is the calculated concentration of either dry matter (DM), ash, organic matter (OM), crude protein (CP), crude fat, sugar, starch, residual fiber (RF), soluble non-starch polysaccharides (sNSP) in the *k*th diet (g kg^-1^ feed or g kg^-1^ DM diet); $n_{k}$ = number of ingredients in the *k*th diet; $a_{jk}$ denotes the proportion of the *j*th feed ingredient in the *k*th diet (kg DM feed ingredient/kg DM diet), and $x_{ij}$ denotes the *i*th concentration of either DM, ash, OM, CP, crude fat, sugar, starch, or RF in *j*th feed ingredient (g kg^-1^ DM feed ingredient). The values for DM, ash, OM, CP, crude fat, sugar, sNSP, and starch concentrations of the individual feed ingredients were obtained from the Danish feed table [10] and Bach Knudsen [11]. The dietary concentration of the RF was calculated by subtracting the concentration of crude fat, CP, starch, and sugar from the OM content.

The average daily feed intake of weaned, grower-finisher pigs, and sows was calculated following the method described in detail by Lashkari et al. [12]. The input values for animal weight, feed intake, animal performance, and reproduction used for the feed intake calculations were obtained from the Danish manure normative system for 2024/2025 [13]. Feed intake of piglets from birth to weaning was quantified as 0.5 feed units per weaned pig, reflecting the typical consumption observed under standard rearing conditions. It was assumed that piglets (from birth until weaning) receive the same diet as weaned pigs to support a smoother post-weaning transition (Table 1).

The nutrient intake was thereafter calculated by multiplying each nutrient’s dietary concentration by the average daily feed intake. The resulting values were then converted to C equivalents using the conversion factors established by Brouwer [14]. The C contributions from the intake of individual nutrients were then summed to calculate total C intake.

### Carbon output

#### Feces.

Fecal C excretion was estimated by converting the concentrations of excreted nutrients to C equivalents and summing the contributions from each nutrient.

The calculation of the OM, CP, crude fat, the sum of starch and sugar, and RF excretion in feces of weaned and grower-finisher pigs and sows is described in detail by Lashkari et al. [12]. Briefly, digested nutrient amounts were estimated by multiplying nutrient intake by the corresponding apparent total tract digestibility (ATTD) coefficient. The ATTD coefficients required for the calculation of OM, CP, and crude fat excretion in feces of weaned and grower-finisher pigs and sows were obtained from the INRAE-CIRAD-AFZ feed tables [15]. The ATTD coefficients for the combined fraction of sugar and starch were obtained from Just et al. [16] and were assumed to be consistent across all pig categories. The calculation of the OM, CP, crude fat, the sum of starch and sugar, and RF excretion in feces of piglets (from birth until weaning) was calculated using the same approach applied to weaned and grower-finisher pigs, employing the same digestibility coefficients.

Fecal excretion was then calculated as the difference between total nutrient intake and the digested fraction. The excreted amounts were then converted to C equivalents using the conversion factors proposed by Brouwer [14], and the resulting values were summed to determine total fecal C output.

#### Urine.

Urinary C output was calculated based on the molecular mass ratio of C to N in urea and non-urea N, assuming that 75% of urine N was excreted as urea N and the remaining 25% as non-urea N, as proposed by Lashkari et al. [12]. Figueroa et al. [17] found that ~30% of non-urea nitrogen in pig urine was creatinine. Hippuric acid and its derivatives associated with the breakdown of phenolics have been identified in pig urine as breakdown products of phenolic compounds [18]. Allantoin has been found in pig urine and is a known breakdown product of purines [19]. To our knowledge, a complete quantitative profile of N-containing metabolites in pig urine is still missing in the literature. Hence, the authors roughly assumed that the non-urea N was a mixture of equimolar creatinine, allantoin, and hippuric acid, which yields an average elemental composition of C_5.67_H_7.33_N_2.67_O_2.33_ (C:N mass ratio of ~1.82), while acknowledging potential variation in metabolite profiles. Although this simplification does not capture the full variability in metabolite composition, it provides a chemically consistent and transparent framework for estimating the associated C content.

Daily urinary N excretion in finishing pigs and sows was estimated using a prediction equation developed by Vu et al. [20]:


$$\begin{array}{c} \text{Urine N }(\text{g}/\text{day})\;=\;-\text{21}.\text{20 }+\;\text{0}.\text{134 }\times \text{ dietary CP }+\text{ 10}.\text{15 }\times \text{ DM intake} \end{array}$$ (2)

where N = nitrogen; CP = crude protein (g kg^-1^ DM); DM = dry matter (kg d^-1^).

However, the predictive equation was not suitable for estimating daily urinary N excretion in piglets (from birth until weaning) and weaned pigs, as it was developed using data from pigs weighing 28–94 kg, which falls outside the weight range for piglets and weaned pigs resulting in systematic overestimation. As a result, it tended to overestimate daily urinary N excretion in piglets and weaned pigs. Therefore, in the case of weaned pigs and piglets, daily urinary N excretion was estimated by subtracting fecal N excretion and N retention from the total N intake as described by Lashkari et al. [12].

#### Exhalation.

The C output from exhalation was calculated by estimating daily CO_2_ production for weaned and grower-finisher pigs, sows, and converting it to C equivalents.

For the calculation of the daily CO_2_ production of piglets, weaned and grower-finisher pigs, the prediction equation of Pedersen [21], as cited by Philippe and Nicks [22] was used:


$$\begin{array}{c} {\text{CO}}_{\text{2}},\text{ pig }(\text{kg }{\text{CO}}_{\text{2}}\;{\text{d}}^{-\text{1}})\;=\;\text{0}.\text{0998 }\times \;{\text{BW}}^{\text{0}.\text{6}\text{4}\text{6}} \end{array}$$ (3)

where, CO_2,_ pig = predicted CO_2_ production (kg CO_2_ d^-1^), and BW = body weight of the pig (kg). The prediction equation was selected because the study dataset included values from weaned pigs starting at 4.9 kg, as well as grower-finishing pigs and sows [21].

The BW of piglets, weaned and grower-finisher pigs used in the equation, was determined as the average of the initial and final weights of the pigs within each pig category that were obtained from the Danish manure normative system [13].

For the calculation of the daily CO_2_ production of sows, the values of 2.23 and 3.68 kg CO_2_ animal^-1^ d^-1^ for gestating and lactating sows, respectively, were used. The values were proposed by Pedersen et al. [23] as cited by Philippe and Nicks [22]. To account for the complete reproduction cycle, the CO_2_ production of gestating and lactating sows was proportionally weighted based on the duration (in days) of each reproductive phase.

The CO_2_ mass (kg animal^-1^ d^-1^) was converted to liters using the gas molar volume at standard temperature and pressure and then converted to C equivalents using the conversion factor of 0.536 g L^-1^ proposed by Brouwer [14].

#### Enteric methane.

The C output through enteric fermentation was calculated in a similar manner to C output through exhalation, where the daily CH_4_ production of piglets, weaned and grower-finisher pigs, and sows was estimated by using a prediction equation and afterwards converted to C equivalents.

For the calculation of the daily CH_4_ production of piglets, weaned and grower-finisher pigs, the prediction equation of Sattarova et al. [24] was used:


$$\begin{array}{c} \text{Enteric }{\text{CH}}_{\text{4}}\;(\text{L }{\text{d}}^{-\text{1}})\;=\;-\text{0}.\text{619 }+\;\text{0}.\text{032 }\times \text{ sNSP }+\;\text{0}.\text{025 }\times \text{ BW} \end{array}$$ (4)

where sNSP = dietary intake of soluble non-starch polysaccharides (g d^-1^), and BW = body weight of the pigs (kg).

The BW of piglets, weaned and grower-finisher pigs applied in the equation was estimated as the mean value of the initial and final weights of the pigs within each pig category as reported in the Danish manure normative system [13].

For the calculation of the daily CH_4_ production of sows, a prediction equation proposed by Jørgensen et al. [25] was used:


$$\begin{array}{c} \text{Enteric }{\text{CH}}_{\text{4}}\;(\text{L }{\text{d}}^{-\text{1}})\;=\;\text{0}.\text{44 }+\;\text{0}.\text{0206 }\times \text{ fermentable fiber intake} \end{array}$$ (5)

where the fermentable fiber intake (kg d^-1^) was estimated by subtracting the digestible fat, CP, sugar, and starch from the digestible OM content, where the digestibility coefficients of starch and sugar were assumed to be 100%.

The daily enteric CH_4_ production of piglets, weaned and grower-finisher pigs, and sows was thereafter converted to C equivalents using the conversion factor of 0.536 g L^-1^ proposed by Brouwer [14].

### Carbon retention

Carbon retention in the animal body was quantified by converting protein and lipid retention into their respective C equivalents and subsequently summing the contributions from each nutrient.

Protein retention in piglets (from birth to weaning), weaned pigs, grower-finisher pigs, and sows was quantified by converting N concentration (g N kg of gain^-1^) obtained from the Danish manure normative system for the year 2024/2025 [13]. The applied N concentrations were 25.7, 30.4, 29.6, and 22.0 g N kg of gain^-1^ for piglets, weaned pigs, grower-finisher pigs, and sows, respectively were converted to protein equivalents using the standard conversion factor of 6.25. Protein retention was subsequently converted to C equivalents using the conversion factor proposed by Brouwer [14].

Lipid retention of weaned and grower-finisher pigs was estimated as 15 and 28.5% of daily BW gain, respectively, based on values reported by de Lange et al. [26] and the National Research Council [27]. Daily gain of weaned and grower-finisher pigs was obtained from the Danish national average productivity data [28]. However, the methodological approach is not limited to input values for the daily gain used in the current study and can be applied to other conditions.

For piglets and sows, lipid retention was estimated using chemical body composition values obtained from an internal, unpublished report from Aarhus University (Fernandez & Danfær, 2007). These composition values were derived from a study involving 183 pigs ranging from birth to adulthood, in which whole-body chemical analyses were conducted to quantify nutrient retention patterns. Based on the chemical body composition data, lipid retention was estimated at 125 g kg^-1^ and 379 g kg^-1^ live weight for piglets and sows, respectively. Daily lipid retention per animal was calculated by multiplying the lipid content per kg of live weight by the corresponding daily BW gain. For piglets, daily BW gain was calculated by multiplying the number of weaned pigs per sow per year by the pigs’ weight at weaning obtained from Sørensen et al. [13] and divided by 365. For sows, the annual weight gain is estimated to be 90 kg [13].

The resulting lipid retention estimates of piglets, weaned and grower-finisher pigs, and sows were subsequently converted to C equivalents using the conversion factor proposed by Brouwer [14].

### Validation

An independent dataset was used to determine whether models applied in the current study provided robust and reliable C balance estimates. The validation dataset originated from an experimental study involving grower-finisher pigs and gestating sows, in which C fluxes were directly quantified. The study design, dietary treatments, animal BW, feed and DM intake, chemical composition of the diets, and all other relevant input parameters are described in detail by Sattarova et al. [29]. However, the analytical methods used to quantify the C balance were not described, and the resulting C balance was not reported in Sattarova et al. [29]. In the underlying experiment, all feed, fecal, and urine samples were analyzed in duplicate for C using analytical methods described in detail in Neergaard et al. [30]. Enteric CH_4_ and CO_2_ production were measured in respiration chambers as described by Jørgensen et al. [31], and multiplied by the conversion factor proposed by Brouwer [14] to convert to C equivalents. In the underlying experiment, C retention was calculated as the difference between C intake and C excreted (urine, feces, CH_4_, and CO_2_), and was therefore not used for the evaluation of the method applied for predicting retained C.

The experimentally determined C balance, excluding retained C, was subsequently compared with the C balance predicted using the methods described in Sections 2.1 and 2.2, with input values for diet composition, animal BW, and feed and DM intake obtained from the experimental study [29].

## Results

The estimated C balance obtained using the different prediction models for weaned pigs, grower–finisher pigs, and sows is presented in Table 2. For grower–finisher pigs and sows, the C balance was not notably influenced by feed production type (home-grown vs. factory-produced). The C exhaled as CO_2_ represented one of the biggest excretion sources, accounting for approximately 70–80% of total C losses across all pig categories examined in the present study. In contrast, C emitted as enteric CH_4_, represented the smallest excretion source, accounting for only 0.2–0.6% of total C losses across all pig categories investigated in the current study. The C retained in the animal body ranged from 96.9 to 328 g d^-1^ across all pig categories. The proportion of C intake retained gradually decreased from weaned to grower-finisher pigs to sows, corresponding to 35, 32, and 12.6%, respectively. For grower-finisher pigs and sows, the amount of unexplained C ranged from 55 to 314 g d^-1^, whereas the amount of unexplained C was negative for weaned pigs (−41.6 g d^-1^), indicating that the predicted C intake was lower than the combined predicted C excretion and retention.

**Table 2:** Carbon (C) balance (g C animal^-1^ day^-1^) calculated for weaned and grower-finisher pigs, and sows including piglets from birth to weaning.

	Pig category				
Item	Weaned pigs	Grower-finisher pigs		Sows^b^	
**Feed production type**	Average^a^	Home-grown	Factory-produced	Home-grown	Factory-produced
**Carbon intake, g d^-1^**	280	1006	1015	1526	1569
**Carbon output, g d^-1^**					
Feces	41.4	158	171	212	239
Urine	2.93	21.4	21.3	28.4	29.2
Enteric methane	0.36	2.36	2.36	5.29	5.82
Exhalation, carbon dioxide	180	437	437	786	786
**Carbon retention, g d^-1^**	96.9	328	328	195	195
**Carbon unexplained, g d^-1^** ^**c**^	−41.6	59	55	299	314
**Carbon unexplained, % of intake**	−14.9	5.9	5.4	19.6	20.0

^a^Average of home-grown and factory-produced feed composition.

^b^C balance of sows includes the C balance of piglets from birth to weaning.

^c^C unexplained represents the difference between C intake and C outputs, including nutrient excretion in urine and feces, losses due to exhalation and enteric fermentation, and nutrient retention in the animal’s body.

**Fig 1:**
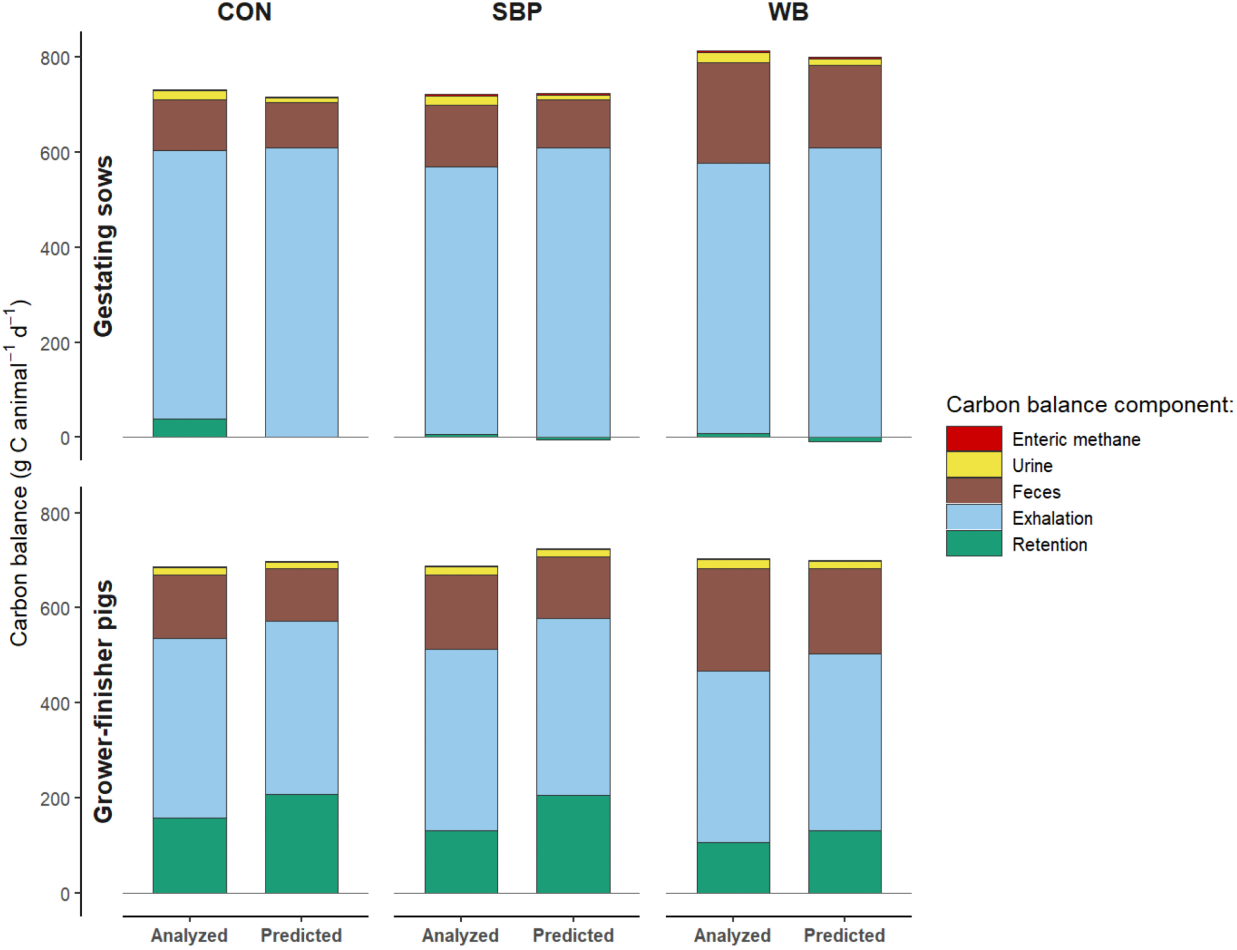
Carbon (C) partitioning in grower-finisher pigs and gestating sows across control (CON), wheat bran (WB), and sugar beet pulp (SBP) diets: analyzed versus predicted values^a^. ^a^ Stacked bars show the contribution of different C fractions (feces, urine, enteric methane, CO_2_ exhalation, and retention) of the total C intake. Retention was calculated as the difference between total C intake and the sum of C outputs (feces, urine, CO_2_ exhalation, and enteric methane), since retention was not directly analyzed.

To validate the applied method for determining C balance using different prediction models, the experimentally determined C intake and excretion data from grower-finisher pigs and gestating sows obtained from the study by Sattarova et al. [29] were compared with model-predicted values (Tables 3 and 4, and Fig 1). The predictions were generated using the same input parameters as in the experiment, including diet composition, feed and DM intake, and animal BW, to assess the accuracy and reliability of the models in estimating C fluxes.

**Table 3:** Comparison of experimentally determined and predicted carbon (C) intake and excretion (g C animal^-1^ day^-1^) in grower-finisher pigs fed diets with varying dietary fiber content^a^.

	Diets					
	CON diet		WB diet		SBP diet	
**Item** ^**b**^	Analyzed	Predicted	Analyzed	Predicted	Analyzed	Predicted
**Carbon intake, g d^-1^**	686	697	703	699	689	725
**Carbon output, g d^-1^**						
Feces	133	110	215	180	158	130
Urine	16	14	20	15	16	14
Enteric methane	1.45	1.37	1.66	1.26	2.59	3.5
Exhalation, carbon dioxide	378	365	360	372	380	372
**Carbon retention, g d^-1^** ^**c**^	158	207	107	131	132	206

^a^CON = control diet; WB = diet high in insoluble fiber content; SBP = diet high in soluble fiber content. For details on the study design, diet composition, nutrient content, animal body weights, and feed and dry matter intake used to predict carbon intake and excretion based on analyzed carbon, see Sattarova et al. [29].

^b^The analyzed mean values for C intake and excretion represent 6 individual observations, respectively.

^c^Calculated as the difference between C intake and output.

**Table 4:** Comparison of experimentally determined and predicted carbon (C) intake and excretion (g C animal^-1^ day^-1^) in gestating sows fed diets with varying dietary fiber content^a^.

	Diets					
	CON diet		WB diet		SBP diet	
**Item** ^**b**^	Analyzed	Predicted	Analyzed	Predicted	Analyzed	Predicted
**Carbon intake, g d^-1^**	732	716	813	791	723	720
**Carbon output, g d^-1^**						
Feces	108	95	213	175	130	102
Urine	18	10	20	13	18	10
Enteric methane	2.6	2.0	3.3	2.7	5.0	3.9
Exhalation, carbon dioxide	564	609	569	609	564	609
**Carbon retention, g d^-1^** ^**c**^	39.4	0.05	7.7	−8.7	6.0	−4.9

^a^CON = control diet; WB = diet high in insoluble fiber content; SBP = diet high in soluble fiber content. For details on the study design, diet composition, nutrient content, animal body weights, and feed and dry matter intake used to predict carbon intake and excretion based on analyzed carbon, see Sattarova et al. [29].

^b^The analyzed mean values for C intake and excretion represent 6 individual observations, respectively.

^c^Calculated as the difference between C intake and output.

Overall, predicted C intake of grower-finisher pigs and gestating sows closely aligned with the analyzed values, except for grower-finisher pigs fed the SBP diet, for which C intake was predicted to be 5% higher than the analyzed values (Tables 3 and 4). Predicted values of C exhaled as CO_2_ in grower-finisher pigs and sows were in close agreement with experimentally measured values, with deviations ranging from −3.3 to 3.6% in grower–finisher pigs and from −7.0 to −8.0% in gestating sows compared to experimentally measured values. Predicted C emissions emitted as enteric CH_4_ in gestating sows and grower-finisher pigs were generally underestimated relative to experimentally measured values, except in grower-finisher pigs fed the SBP diet. Predicted C excretion in the feces of grower-finisher pigs and gestating sows was predicted with similar deviation compared to the analyzed values, consistently underestimated by around 18% across all dietary treatments. Predicted urinary C excretion in grower-finisher pigs was underestimated to a similar extent as fecal C excretion, whereas the urinary C prediction for gestating sows exhibited poor accuracy, indicating that the model used to estimate urinary C excretion in gestating sows was inadequate.

The C retention in the animal body was not measured experimentally, but estimated as the difference between C intake and C output. In grower-finisher pigs, calculated C retention was, on average, 49 g d^-1^ higher across all dietary treatments relative to the experimental values. In contrast, in gestating sows, calculated C retention was, on average, 22.2 g d^-1^ lower across all dietary treatments compared with the experimental values.

## Discussion

The aim of the study was to establish and provide a theoretical basis for C mass balance for weaned pigs, grower-finisher pigs, and sows based on predicted dietary C intake and C output via excretion, gas emissions, and body tissue retention. Overall, the C mass balances calculated by using different prediction equations fell within the range reported for grower-finisher pigs [32–34] and, to a lesser extent, for sows [35]. The deviation relative to Wisbech et al. [35] likely reflects differences in simulated feed intake and physiological status, as the sows in the experiment were non-lactating and non-gestating, whereas the present study considered sows over an entire annual production cycle.

In the current study, the C intake of weaned and grower-finisher pigs and gestating sows was estimated by converting calculated dietary nutrient intake into C equivalents. The C intake, irrespective of production stage, is difficult to compare across published studies because it is determined by a specific combination of diet composition, nutrient concentrations, feed intake, and animal BW. Consequently, a specific combination of dietary and animal characteristics makes it challenging to identify studies with sufficiently comparable characteristics for direct comparison. However, predicted C intake of grower-finisher pigs and gestating sows estimated by converting calculated dietary nutrient intake into C equivalents was closely aligned with the experimentally measured C intake (Tables 3 and 4), indicating that estimating C intake through conversion of calculated nutrient intake into C equivalents is a valid and reliable approach.

Predicted C exhaled as CO_2_ constituted the greatest predicted C output source, accounting for 70–80% of total predicted C output or 43–62% of predicted C intake across all pig categories investigated in the present study. The proportion of C exhaled relative to the C intake in grower-finisher pigs was comparable to values reported in previous studies, ranging from 48 to 61% [32,33,36,37]. Moreover, in the experiment used to validate the C balance equations for grower-finisher pigs (Table 3), the proportion of analyzed C exhaled as CO_2_ relative to intake ranged from 51 to 55%, closely matching the predicted C exhaled. These results indicate that the prediction equation used for CO_2_ production is accurate and robust for estimating C exhaled in grower-finisher pigs.

In the present study, the predicted C exhaled relative to the predicted C intake of sows over the entire annual production cycle was estimated at 50%. In contrast, for non-gestating and non-lactating sows, C exhaled as a percentage of C intake ranged from 53 to 81% across dietary treatments, with an overall average of 70% across all diets [35]. Moreover, in the experiment used to validate the C balance equations (Table 4) for gestating sows, the proportion of C exhaled ranged from 70 to 78%. The differences compared to the calculated exhaled C likely reflect variation in production stage, comparing non-gestating and non-lactating with gestating sows or with sows averaged over an entire annual production cycle. In the current study, the daily CO_2_ production of sows was estimated using constants 2.23 and 3.68 kg CO_2_ animal^-1^ day^-1^ for gestating and lactating sows, respectively, proportionally weighted according to the duration of each reproductive phase. Although using fixed constants may not be the most precise approach, it remains reliable, as validation (Table 4) showed that predicted C from CO_2_ exhalation of gestating sows systematically overestimated experimentally measured values by no more than 8%.

Prediction of C in urine and feces was more challenging, as it partially depends on the amount of N excreted and the repartition of N between urine and feces, which may vary with dietary treatment [32,38]. Predicted C excretion in urine and feces relative to the C intake was 15.8% in weaned pigs, 18.4% in grower-finisher pigs, and 16.4% in sows. The proportion of C excreted in urine and feces in grower-finisher pigs was consistent with values reported in previous studies with grower-finisher, ranging from 12 to 25% depending on the dietary treatment [32–34,37,39]. In a separate study with non-lactating and non-gestating sows, urine and fecal C excretion relative to the C intake varied from 19–58% across all investigated dietary treatments, indicating that more variation in predicted fecal C excretion can be expected, especially with inclusion of feed ingredients with high content of insoluble fiber [34,35].

In the experiment used to validate the C balance equations, experimentally measured C excretion in urine and feces accounted for an average of 26.8% of total C intake in grower–finisher pigs and 22.1% in gestating sows, whereas the corresponding predicted values averaged 21.9% and 18.0%, respectively, underestimating C excretion in urine and feces by 18% in both groups. Jørgensen et al. [34] reported a similar pattern, in which calculated fecal C excretion of grower-finisher pigs was underestimated by 2% or 11% relative to experimentally measured values, depending on the prediction approach. The accuracy of nutrient digestibility coefficients used to predict nutrient excretion may be influenced by the repeatability of the digestibility coefficient estimates [40], which in turn affects the prediction of C excretion in urine and feces of pigs.

A related limitation concerns the assumption of additivity when estimating fecal C output using ingredient-level ATTD coefficients. In the model, fecal C excretion of each nutrient is derived from ATTD coefficients of individual ingredients, combined with ingredient-specific nutrient concentrations and dietary inclusion rates. However, this method assumes additivity of digestibility values and does not account for potential interactions among ingredients within mixed diets. Moreover, although the use of ATTD coefficients is justified because basal endogenous C losses must be included as in the case when ATTD values are used, it should be acknowledged that ATTD values of individual ingredients are not inherently additive in mixed diets. Numerous digestibility studies have shown that such interactions are common in practical feeding scenarios and can significantly influence nutrient degradation, endogenous losses, and fecal output [41–43]. Because the present model does not incorporate these interaction effects, predictions of fecal C output may be subject to systematic bias when diets contain ingredients with known interaction potential. Acknowledging this limitation is essential, and future model refinements should integrate mechanisms or correction factors that account for ingredient interactions to improve the accuracy of fecal C predictions.

Enteric CH_4_ production is well documented as higher in adult animals than in younger pigs, reflecting age-related differences in digestive physiology and microbial fermentation capacity [29,44,45]. This pattern is also evident in the present study, as predicted C ouput from enteric CH_4_ production increased from weaned to grower-finisher pigs and further to sows (Table 2). Overall, the C output from enteric CH_4_ production accounted for a very small proportion of total C intake, ranging from 0.16% in weaned pigs to 0.38% in grower-finisher pigs, and 0.53% in sows (Table 2). In the experiment used to validate the C balance equations, experimentally measured C output from CH_4_ averaged 0.27% of C intake in grower-finisher pigs, and 0.48% in gestating sows, whereas the corresponding predicted values were 0.28% and 0.38%, respectively. Although the relative deviation between predicted and measured C values was considerable, the absolute discrepancy remained small, amounting, on average, to only 0.46 g d^-1^ in grower-finisher pigs and 0.77 g d^-1^ in gestating sows (Fig 1). This suggests that, although the prediction equations could be improved, the amount of C involved is so small that such refinements are unlikely to noticeably affect the total C balance.

Data on body composition in terms of C in weaned pigs, grower-finisher pigs, and sows are scarce. However, predicted C retention relative to the C intake in grower-finisher pigs was very close to the values found in previously published studies [32,33,37]. In the present study, C retention was estimated from predicted lipid and protein retention using established constants and subsequently converted to C equivalents. Overall, the constants applied for the calculation of protein and fat retention in piglets, weaned, and grower-finisher pigs, and sows were broadly consistent with values reported in literature [26,46–51]. However, protein and fat retention may be affected by breed, feeding regime, health status, and environmental conditions, implying that greater variation in predicted C retention is expected when applying the approach used in the present study. Additionally, future studies should focus on directly measuring C retention by analyzing whole-body composition at different ages, rather than relying solely on a difference-based approach (C intake – C output) or on predictions derived from protein and fat retention. Direct analytical measurements would provide more accurate estimates of C balance and substantially reduce the uncertainty associated with the predictive approach used in the present study.

The C balance in weaned pigs represents an uncertainty in the model, as data on weaned pigs are scarce. Moreover, no experimental data were available to validate the accuracy of the predictions for this category, indicating that the currently used prediction equation may require refinement.

Rearing conditions such as stocking density, housing system, environmental enrichment, and thermal and hygienic environments are known to influence feed intake, metabolic efficiency, and stress physiology in pigs, all of which can influence nutrient partitioning. In addition, physiological stressors and health status—such as weaning stress, reproductive burden (gestation and lactation), social competition, and subclinical disease — can further affect nutrient digestibility and energy metabolism. Although these variables were not parameterized due to the lack of quantitative data directly linking them to C balance, they may contribute to unexplained variability and should be recognized as a source of potential confounding when interpreting model outputs.

A remaining limitation of the present model is the relatively high proportion of unexplained C, particularly in weaned pigs and sows. This likely reflects the accumulation of small prediction errors across multiple model components, including feed intake estimation, digestibility coefficients, and partitioning of C between exhaled CO_2_, excreta, and body retention. In weaned pigs, the higher unexplained fraction may be attributed to the limited availability of experimental data for this category and to their highly variable physiology during the post-weaning transition, characterized by fluctuating feed intake, immature digestive function, and rapidly changing nutrient utilization. These factors increase uncertainty in predicting digestibility, excretion, and metabolic CO_2_ production. For sows, the elevated unexplained C fraction likely reflects the aggregation of different physiological stages (gestation, lactation, including maintenance) within the annual production cycle, each associated with distinct metabolic rates, feed intake patterns, and body reserve mobilization. The use of fixed coefficients for CO_2_ production and simplified assumptions for body tissue retention across these stages may therefore introduce systematic bias.

In addition, the prediction equations applied in the present study were primarily derived from datasets generated under specific experimental conditions and animal categories, which may limit their applicability across broader physiological states and modern production systems. Many of these equations rely on generalized constants or average digestibility values that may not fully capture variation due to diet composition, ingredient interactions, genetics, or health status. As a result, structural limitations of the existing models may contribute to systematic over- or underestimation of individual C fluxes, which cumulatively appear as unexplained C. Updating these prediction equations using larger, more diverse datasets and incorporating dynamic, stage-specific parameters would likely reduce the residual imbalance and improve the robustness of C mass balance estimations. Overall, while each source of error is likely small, their combined effect can explain the relatively high unexplained C observed in weaned pigs and sows. In addition to limitations associated with the prediction equations, uncertainties inherent to the validation dataset should also be acknowledged. Deviations between predicted and observed C fluxes likely reflect the accumulation of small errors across multiple model components, including feed intake estimation, digestibility coefficients, and the partitioning of C among exhaled, CH_4_, CO_2_, excreta, and body retention. These inconsistencies are further compounded by the use of equations and established constants originally developed for separate purposes, which may not be fully compatible within a unified C balance framework. Collectively, these factors may contribute to the residual unexplained C observed in the validation.

## Conclusions

The present study proposes a theoretical framework for estimating C mass balance in weaned pigs, grower-finisher pigs, and sows by predicting C intake and partitioning C outputs via excretion, gas emissions, and body tissue retention. Overall, the predicted C mass balances showed reasonable agreement with published and experimental data, particularly for grower-finisher pigs and, to a somewhat lesser extent, for sows. The results of the present study demonstrates that estimating C intake via nutrient-to-carbon conversion and predicting major C output pathways using established equations may provide a useful basis for approximating C flows across production stages. However, validation against experimental data indicated that there is good accuracy for C intake and C output through exhalation, whereas C output in feces, urine, enteric CH_4_, and, especially, C retention remain uncertain and require further refinement.

## Abbreviations

ATTD: apparent total tract digestibility
BW: body weight
C: carbon
CH_4_: methane
CO_2_: carbon dioxide
CP: crude protein
DM: dry matter
GHG: greenhouse gas
N: nitrogen
OM: organic matter
RF: residual fiber
sNSP: soluble non-starch polysaccharides
